# The Role of miRNA in the Pathophysiology of Neuroendocrine Tumors

**DOI:** 10.3390/ijms22168569

**Published:** 2021-08-09

**Authors:** Lukas Geisler, Raphael Mohr, Joeri Lambrecht, Jana Knorr, Henning Jann, Sven H. Loosen, Burcin Özdirik, Tom Luedde, Linda Hammerich, Frank Tacke, Alexander Wree, Teresa Hellberg, Christoph Roderburg

**Affiliations:** 1 Department of Hepatology and Gastroenterology, Campus Virchow Klinikum (CVK) and Campus Charité Mitte (CCM), Charité Universitätsmedizin Berlin, Augustenburger Platz 1, 13353 Berlin, Germany; lukas.geisler@charite.de (L.G.); raphael.mohr@charite.de (R.M.); joeri.lambrecht@charite.de (J.L.); jana.knorr@charite.de (J.K.); henning.jann@charite.de (H.J.); burcin.oezdirik@charite.de (B.Ö.); Linda.Hammerich@charite.de (L.H.); frank.tacke@charite.de (F.T.); alexander.wree@charite.de (A.W.); teresa.hellberg@charite.de (T.H.); 2 Clinic for Gastroenterology, Hepatology and Infectious Diseases, University Hospital Düsseldorf, Medical Faculty of Heinrich Heine University Düsseldorf, Moorenstraße 5, 40225 Düsseldorf, Germany; sven.loosen@med.uni-duesseldorf.de (S.H.L.); Tom.Luedde@med.uni-duesseldorf.de (T.L.)

**Keywords:** NET, miRNA, biomarker, prognosis, diagnosis

## Abstract

Neuroendocrine tumors (NETs) represent a tumor group that is both rare and heterogeneous. Prognosis is largely determined by the tumor grading and the site of the primary tumor and metastases. Despite intensive research efforts, only modest advances in diagnostic and therapeutic approaches have been achieved in recent years. For patients with non-respectable tumor stages, prognosis is poor. In this context, the development of novel diagnostic tools for early detection of NETs and prediction of tumor response to therapy as well as estimation of the overall prognosis would greatly improve the clinical management of NETs. However, identification of novel diagnostic molecules is hampered by an inadequate understanding of the pathophysiology of neuroendocrine malignancies. It has recently been demonstrated that microRNA (miRNA), a family of small RNA molecules with an established role in the pathophysiology of quite different cancer entities, may also play a role as a biomarker. Here, we summarize the available knowledge on the role of miRNAs in the development of NET and highlight their potential use as serum-based biomarkers in the context of this disease. We discuss important challenges currently preventing their use in clinical routine and give an outlook on future directions of miRNA research in NET.

## 1. Introduction

Neuroendocrine tumors (NETs) constitute a highly heterogeneous group of malignancies arising from cells of the disseminated neuroendocrine system, with variable prognosis and tumor biology. They may occur in any part of the body but are most commonly found in the gastrointestinal tract, the lung, or the pancreas. Although a rare disease, the incidence of NET has been increasing in recent decades [1,2,3].

Based on the World Health Organization’s (WHO) classification, NETs are characterized by the grade of neuroendocrine differentiation and their proliferative index (Ki-67) from G1 to G3 (<3%, 3–20%, >20%). Prognosis of NET mainly depends on early diagnosis. However, many patients are diagnosed in advanced disease stages where curative treatment options are lacking. In order to improve early diagnosis, intensive research activities have been undertaken trying to identify novel and reliable biomarkers for clinical routine. Being a rare and diverse group of tumors, large cohorts allowing a systematic identification of markers are lacking [4]. In this context, chromogranin A (CgA) certainly is the marker with the highest specificity and sensitivity in NET diagnosis. Nevertheless, it rather reflects tumor response to treatment and only has limited value in the initial diagnostic process [5,6]. Just recently, the NETest, a novel blood multigene RNA transcript assay, was described and validated as a diagnostic test for NETs of different localizations [7,8]. MicroRNAs (miRNAs) are short, conserved non-coding RNAs that do not contain information to encode for proteins but are involved in post-transcriptional gene regulation and silencing [9]. By regulating whole networks of genes, miRNAs are involved in manifold physiological and pathophysiological processes, including cell cycle regulation, cellular differentiation, survival, metabolism, and immune cell regulation [10]. Due to their ability to act as master regulators of multiple genes, they are expected to play a significant role in cancer development, homeostasis, and possibly immune escape [11]. Besides their role in the regulation of gene expression, miRNAs have been proposed as diagnostic, prognostic, and predictive biomarkers in several human diseases, including various cancers. Several reports described alterations of miRNA expression levels in different tumor entities, highlighting the deep integration of miRNAs in the pathophysiology of cancer [12,13,14].

miRNAs are involved in different aspects of the pathophysiology of NETs and thus have the potential to serve as diagnostic and predictive biomarkers in the context of these tumors [15]. In this review, we summarize the available knowledge of the role of miRNAs in the development of NETs. We discuss important challenges currently preventing their use in clinical routine and give an outlook on future directions of miRNA research in NET.

## 2. Biogenesis and Function of miRNAs

miRNAs are defined as small, single-stranded RNAs with an average length of 22 nucleotides. They do not encode for proteins but negatively regulate the expression of their target genes on a post-transcriptional and post-translational level [16,17,18]. About 50% of the human transcriptome is controlled by miRNAs, and in silico data predicted that more than 45,000 miRNA target sites are present in human DNA [19,20].

Human miRNA biogenesis is a multistep process composed of four phases, with both nuclear and subsequent cytoplasmic cleavage events. RNA polymerase II and III process primary miRNAs (pri-miRNAs) of 500–3000 nucleotides in length, which can be derived from introns or from long non-coding RNAs (lncRNAs). In the nucleus, pri-miRNAs are turned into pre-miRNA hairpin precursors by the microprocessor complex, consisting of the RNA binding protein DiGeorge Syndrome Critical Region 8 (DGCR8) and a ribonuclease III enzyme called Drosha. The generated pre-miRNAs are exported to the cytoplasm in an Exportin5/RanGTP-dependent manner and then cleaved by the RNase III endonuclease Dicer into ~22-nucleotide-long, double-stranded miRNAs [17,21,22]. One strand of the mature miRNA is loaded into the Argonaute (AGO) family of proteins (AGO1-4 in humans) [23] to form an miRNA-induced silencing complex (miRISC), while the complementary strand is excluded and subsequently degraded.

Depending on the level of sequence complementarity between the miRNA and target messenger RNA (mRNA), the mature miRNA then guides RISC-induced mRNA down-regulation through translational repression, mRNA destabilization, or cleavage [24,25]. To suppress or to fine-tune protein expression, miRNAs interact with the 3′ UTR or with the 5′ UTR of target mRNAs, but interactions with coding sequences and gene promoters have also been reported [24,25]. One miRNA is able to influence hundreds of genes and multiple miRNAs can regulate the same gene by binding neighboring target sites, which can result in cooperative repression.

Many of these master regulators are involved in the regulation of critical molecular processes and are associated in the pathophysiology of a broad range of human diseases, including genetic disorders, inflammatory diseases, and various cancers. It was shown that miRNAs present altered expression patterns in malignant tissues, and miRNA alterations have an association with all major cancer pathways. Recently, different preclinical studies have analyzed the expression patterns of miRNA in gastroenteropancreatic NET, showing that several miRNAs are specifically regulated in the context of NET and might regulate critical steps in the development of these tumors [26,27,28,29,30,31,32,33,34].

miRNA-related research received a stimulating boost when miRNAs were detected in cell-free environments. Changes in the expression profile of circulating miRNAs have been shown to have specific patterns in relation to different cancer types and cancer stages and could provide exceptional potential as biomarkers, particularly in NETs.

## 3. Circulating miRNAs

**Figure 1:**
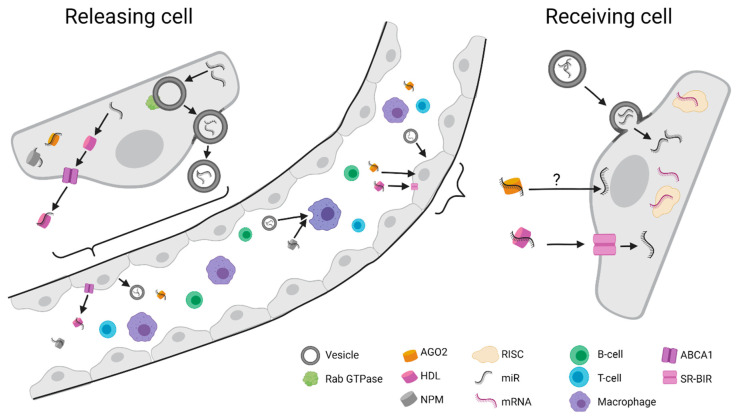
Exemplary transmission of miRNA from donor to acceptor cell: Secretion of miRNAs through complexing with argonaute-2 (AGO2), nucleophosmin (NPM), and high-density lipoprotein (HDL) and through phospholipid-transporting ATPase ABCA1. Microvesicles are released through external budding and plasma membrane fission in association with Rab GTPase and may carry sugars, lipids, protein, DNA, mRNA, and miRNAs. The uptake of HDL-conjugated miRNAs is mediated through HDL-(scavenger)-receptor (SR-BI). The internalization mechanism of AGO2 and NPM conjugates remains unclear. Microvesicles may transmit their load cell-type-dependently via plasma membrane fusion (merging) or endocytosis (receptor-mediated), micropinocytosis (cell-drinking), and phagocytosis (active engulfment). Loading of miRNA to the RISC complex in the target cell will affect target downregulation through translational repression. Figure created with BioRender.com.

There is much evidence that the release of miRNAs is a regulated process, although some extracellular miRNAs are released as by-products of cell injury or cell death [35]. Extracellular miRNAs have been detected in the peripheral blood circulation and other biological fluids, such as urine, tears, colostrum, peritoneal fluid [36], saliva [37], breast milk [38], cerebrospinal fluid [39], and gastric juices [40], and can be used as biomarkers for a variety of cancers. Currently, there are several models that explain the release of miRNAs from the secreting cell, which allows serum stability, correct targeting, and uptake (Figure 1). Four essential mechanisms are currently known, including release within extracellular vesicles [41], association with high-density lipoprotein (HDL) [42], and complexing with protective proteins, such as AGO2 [43] or RNA-binding protein nucleophosmin (NPM1) [44]. The protein–miRNA complex is extremely stable in body fluids such as serum samples and is commonly secreted into the plasma for transport [44,45]. HDL-dependent secretion appears to be regulated by nSMase2 activity and ceramide synthesis, while the ATP-binding cassette transporter A1 (ABCA1) likely mediates the efflux of complexed miRNA. Specific to HDL export is the high affinity for the secretion of miR-223 as well as miR-135. Initially, NPM1 was thought to be one of the essential shuttles of RNA between the nucleus and the cytosol, before studies showed that this nucleolar multifunctional protein also occurs outside of cells and stabilizes miRNA extracellularly [44,46]. Research focusing on AGO2 suggested that there could be cell-specific differences for miRNA secretion, such as in miR-122, which appears to be specific for protein carriers but not extracellular-vesicle-dependent export. While larger microvesicles (up to 1000 nm in size) are commonly released through external budding and plasma membrane fission, smaller extracellular vesicles (approximately 40–100 nm) are usually derived from the fusion of multivesicular bodies (MVBs) with the plasma membrane [47]. Those may additionally carry a load containing sugars, lipids, and protein as well as DNA, mRNA, and miRNAs [48].

Carrying a GGAG motif or post-transcriptional 3′ end uridylation appears to benefit the sorting of miRNA into extracellular vesicles [49]. The release of exosomes is mediated by Rab GTPases, including RAB11 and RAB35, or RAB27A and RAB27B, which facilitate the membrane fusion processes [50]. The uptake mechanisms of this extracellular miRNA are not well-defined. It is suspected that specific receptors on the cell surface take up vesicle-free secreted miRNAs—e.g., HDL-associated miRNAs are taken up by HDL receptor and scavenger receptor BI (SR-BI) [42]. Uptake of vesicle-associated extracellular miRNAs commonly occurs through plasma membrane fusion or endocytosis, including micropinocytosis and phagocytosis [22,51]. Since their discovery, there has been exponentially increasing information about the significant roles of extracellular vesicles in the communication of cells in immunology and tumor biology. Secretion of exosomes can mediate paracrine signals from cancer cells that promote growth in the tumor microenvironment by inhibiting the antitumor immune response and facilitating angiogenesis, cell migration, and metastasis [52]. However, tumor cells, such as gastric cancer cells, were also found to eliminate tumor-suppressor miRNAs by secreting exosomes to maintain their oncogenesis [53].

Due to their stability and resistance to endogenous RNase activity and their ability to reflect the homeostatic response of the organism as well as to be a sign of disease progression, circulating miRNAs are an important tool for the diagnosis and prognosis of NETs and could significantly expand the limited therapeutic arsenal.

## 4. miRNA in the Pathophysiology of NET

### 4.1. General Considerations

**Figure 2:**
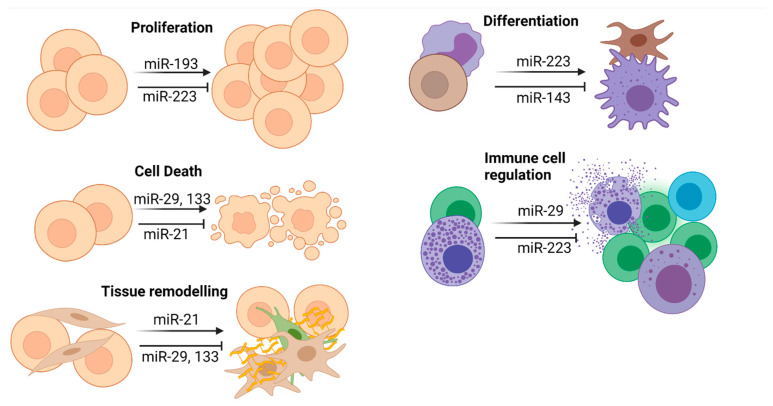
RISC/miRNA-dependent translational repression may induce or inhibit various oncogenic key functions, including proliferation, differentiation, cell death, immune cell regulation, and tissue remodeling/metastasis. Figure created with BioRender.com.

Only recently, different preclinical studies have analyzed the expression patterns of miRNA in gastrointestinal and pancreatic NETs, showing that several miRNAs are specifically regulated in the context of NET and might regulate critical steps in the development of these tumors (Figure 2, Table 1). Thus, a panel of eight miRNAs was shown to be consistently expressed in tissue of gastrointestinal NETs, but with varying levels in the different tumor grades. Among these miRNAs, miR-96-5p was progressively more highly expressed from grade 1 to grade 3; inversely, expression of its target FoxO1, which is involved in the cell cycle and cell death, decreased from grade 1 to grade 3, providing a use case of how miRNAs might be involved in the pathophysiology of NET [54]. Along with miR-96-5p, other miRNAs showed a negative (miR-22, miR-29a, miR-29b, miR-29c, miR-367*, miR-504, miR-513C, miR-1200) or a positive (miR-18a, miR-15b*, miR-335*, miR-1201) correlation to the grade of tumor biology [55]. Many of these miRNAs are involved in the regulation of tumor-related cell functions. As such, miR-29a was found to inhibit growth, migration, and invasion of melanoma A375 cells by directly targeting B lymphoma Mo-MLV Insertion region 1 polycomb ring finger (BMI1), which has been described as an important driver of malignant transformation in many carcinomas [56,57] and is overexpressed specifically in low-grade NETs of the lung. In addition, gastrointestinal neoplasms showed a strict downregulation of miR-133 in metastasis against a primary tumor, hinting at a possible role of miR-133 in regulating invasiveness [26,27,28]. Complementary miR-143 expression was reduced in gastrointestinal neoplasms compared with further downregulation-derived metastasis [29]. Our own data provided initial evidence of the downregulation of miR-223 [30] in the serum of NET patients, without correlating with disease severity or survival. Interestingly, evaluation of miR-21 in pancreatic NETs (pNETs) found a strict correlation with proliferation index and liver metastasis [31], while other studies did not identify any correlation [32]. Similarly, miR-193b was also found to be upregulated in pNET tissue as well as in serum, hinting towards its essential role in disease progression [32]. The inverse correlation between miR-375 and YAP appeared to be essential to neuroendocrine lung xenograft differentiation and proliferation [33], while low levels of miR-34a appeared to benefit the invasiveness of pituitary adenomas, possibly due to increased Survivin (BIRC5) and FGF2 [34].

In addition, different analyses recently demonstrated that treatment with Somatostatin analogues might alter miRNA expression in gastrointestinal NETs. Bosch et al. analyzed for the first time the individual effect of therapy with SSAs on the miRNA dysregulation pattern in small intestinal NENs (SI-NENs), revealing that let-7c-5p was consistently upregulated upon SSA treatment whereas miR-3137 was consistently downregulated [109]. The crucial role of the let-7 family in the carcinogenesis of NETs was demonstrated by Zimmermann et al. and Døssing et al., showing that downregulation of let-7c is involved in the development of NET metastases through targeting of HMGA2, BACH1, and MMP1 [110,111]. Other mechanisms regulated by let-7 in NETs include the regulation of insulin sensitivity, glucose metabolic pathways, and autophagy, highlighting the deep integration of miRNAs in the pathophysiology of NETs [112,113].

### 4.2. miR-21

miR-21, one of the most prominent and abundant regulatory RNAs, is encoded in chromosome 17 within the locus of the vacuole membrane protein 1 [114]. Possible post-transcriptional processing of pri-miR-21 is moderated through TGF-β and downstream activation of Drosha in complex with RNA helicase p68 [115]. Culture of cardiomyocytes in hypoxic conditions with hypoxia-inducible factor 1α (HIF-1α) [116] induces miR-21 expression, alongside the growth factor VEGF, inducible nitric oxide synthase (INoS), and heat shock protein 70 (HSP70) [117]. Cell-specific evaluation of miR-21 expression in vivo could show its ubiquitous presence at higher levels in macrophages, monocytes, and dendritic cells [118]. Interferon presence is positively correlated to miR-21 expression in murine fibroblasts, as signal transducer and activator of transcription 3 (STAT3) as well as nuclear factor kappa-light-chain-enhancer of activated B cells (NF-κB)/p65 bind the miR-21 promotor region [119]. Downregulation of phosphatase and tensin homolog (PTEN) in HCC [120] and simultaneous increased activation of the p85 subunit of phosphoinositide 3-kinase (PI3K) [119] through miR-21 conciliate to increase protein kinase B (AKT) signaling, thus limiting apoptosis signaling and promoting migration/invasion by indirectly inducing matrix metalloproteinases (MMPs). Yang and colleagues identified F-box only protein 11 (FBXO11), which promotes B-cell lymphoma 6 protein (BCL-6) ubiquitylation and consequent degradation in vitro, therefore inhibiting cell proliferation and inducing cell death through apoptosis [121] to be inversely correlated to miR-21 expression [122].

Due to its role in cardiovascular disease and other infections, including hepatitis C and dengue viruses, multiple sclerosis, or pulmonary disease, miR-21’s properties as a standalone diagnostic biomarker for NET are very limited. However, given its functional importance, miR-21 is considered a prototypical oncogene. In pancreatic cancer, increased levels of miR-21 correlated with significantly decreased survival [123]. Similarly, high levels of miR-21 coincided with the presence of glioblastoma when compared with healthy controls but significantly deviated from similar conditions such as meningioma or pituitary adenoma [124]. miR-21 plasma values were significantly altered after treatment and showed a correlation to histopathology. In a similar fashion, miR-21 was upregulated in lung cancer patients compared to healthy controls, while other miRNAs, such as miR-155 and -197, showed a greater specificity as biomarkers for prognosis [125]. In agreement with this, serum miR-21 is also increased in colorectal cancer, while miR-92a for example had a better prognostic value for disease progression [126]. While there is ample evidence for the oncogenic implications of miR-21 upregulation, there is little evidence regarding NET. Levels of miR-21 in a heterogeneous NET cohort were not significantly regulated compared to those in healthy controls [30]. In a more homologous group of pancreatic NETs, expression of serum miR-21 correlated with Ki-67 index and the presence of liver metastasis [31]. Contrastingly, evaluation of pNETs in alternate studies did not reveal any correlation, without considering metastasis [32]. Homogenization of sample evaluation would be necessary to identify a possible role of miR-21 in NETs, while relevant data in this regard remain sparse to this day.

### 4.3. miR-29a

The miR-29 family of RNAs consists of three members, including miR-29a/-29b and -29c encoded on chromosomes 1 and 7, respectively. They are involved in multiple processes and cellular pathways, e.g., the lymphoid cellular immune response, apoptosis, collagen and fibrous matrix deposition, and intestinal epithelial cell homeostasis. While several targets of miR-29 are known to date, several detailed analyses have tried to elucidate the pleiotropic function in regard to lymphoid cells and immune responses. B cells rely on miR-29a expression for the proliferation in germinal centers and consequential secretion of antibodies [127]. In addition, miR-29a promotes a Th17 response by dampening soluble sST2 (IL-33 receptor) transcription [128,129,130]. Multiple major pathways, including the Wnt/β-catenin signaling [131], TGF-b/SMAD3 [131], and fibrogenic pathways, affecting collagens Col1a1, Col1A2, and Col3A1; fibrillin (FBN1), and elastin (ELN1), appear to be regulated through miR-29 in murine models of fibrosis [132,133]. In addition, miR-29 appeared to increase the rate of apoptosis in leukemia [134], hepatocellular carcinoma (HCC) [135], gastric cancer [136], lung cancer [137], and glioblastoma [138] cases through signaling via BCL-2 or myeloid leukemia cell differentiation protein 1 (MCL-1). Apoptosis regulation mediated by miR-29c and the transcription factor C-Jun in endometrial cells has been described. In human breast cancer, those groups of proteins are effectors of regulated cell death signaling, likely upstream of cytochrome C/caspase function, thereby affecting apoptosis directly [139]. In vitro studies also showed that miR-29 activates the p53 pathway and induces apoptosis via suppression of CDC42 [140]. In this context, analysis of miR-29 targets suggests that CDC42 is probably involved in the pathway, potentially leading to colorectal cancer (CRC) development [141]. The regulatory role of miR-29 has also been reported to limit lysyl oxidase-like 2 protein (LOXL2) in squamous cell carcinoma [142]. In addition, tristetraprolin (TTP), an RNA-binding protein and critical player in regulating pro-inflammatory immune responses, has also been identified as a target of miR-29a. Downregulation of TTP mediated by miR-29a is associated with metastasis in breast cancer [143] and pancreatic cancer growth [144]. On a similar note, reduced functioning of the extracellular matrix remodeling MMP2 was shown in ovary [145], gastric [146], and pancreatic cancers [147] and may be regulated through targeting of the Wnt/β-catenin signaling [148]. Studies evaluating CRC models found a straight correlation between miR-29a and MMP-2 promoting the formation of metastasis [149]. Quite interestingly, miR-29a in serum appears to be a potent biomarker for colorectal liver metastasis [150], while total miR-29 family expression, especially higher miR-29b expression in tissue, predicts better overall survival [151]. Aside from the aforementioned pathways, miR-29 also appears to significantly affect and mediate glucose metabolism in ovarian cancers by targeting AKT (protein-kinase B) and through the Warburg effect [152]. In a previous study, upregulation of neuroendocrine CgA was found to be increased in lung squamous cell carcinoma cell lines [153]. Again, these cells were sensitive towards AKT/PI3K/mTOR inhibition, showing that some findings from alternate tumor entities do indeed translate to NET. This was additionally proven by Özdirik et al., as an evaluation of serum miR-29b in patients showed a similar correlation with chromogranin A levels in NET, while not corresponding with Ki-67 index, tumor grading, TMN stage, or overall survival [154].

### 4.4. miR-133

miR-133 is encoded on chromosomes 6, 18, and 20 in its major variants, miR-133a-1, miR-133a-2, and miR-133b. In connection with miR-1 and miR-206, termed the family of myomiRs, initial studies were able to provide a major body of evidence for its function in muscle development and differentiation through Hippo/Notch signaling (HEYL/ HRT2) and transcription factors NR4A and PAX7 [155]. Recent data have evaluated functional implications of the myomiRs in cancer development. Most strikingly, miR-133 interferes with death receptor 5 (DR5), thereby limiting TNF-related apoptosis-inducing ligand (TRAIL)-mediated induction of apoptosis in colon cancer cells [156]. Furthermore, evaluations involving gastric cancer and lung cancer models could show a direct interaction with MCL1 and BCL proteins. Regulation of pro-survival proteins marks miR-133 as a classical tumor suppressor in vitro and in vivo by inducing apoptosis [157,158]. On the same note, overexpression of miR-133 in murine gastric cancer models significantly reduced the expression of cyclin D1 and MMP-9, limiting cellular proliferation, migration, and invasion through targeting of the transcription factor SP1 [159]. Along these lines, assessment of miR-133 in colorectal cancer and osteosarcoma found the activation of major oncogenic pathways, including PI3K, AKT, and ß-catenin signaling [160,161,162]. While there is initial evidence of miR-133 targeting the pyruvate kinase muscle (PKM) group of proteins, which mediate glycolysis and Warburg effects in solid tumors [163], its involvement with epidermal growth factor receptor (EGFR) signaling is well-established. As a major regulator of MAPK/STAT/PI3K signaling [163], EGFR appears to be of major importance by being involved in fundamental processes, including apoptosis, proliferation, migration, adhesion, and invasion in vitro and in vivo [164,165,166,167]. The latter holds true for renal cell carcinoma as well as glioblastoma, where miR-133 was found to affect cellular migration and invasion through MMP-9 and MMP-14 [168,169]. Quite strikingly, the available data show that miR-133 is downregulated in metastasis against a primary tumor, e.g., liver metastasis of small bowl tumor [27], lymph node and liver metastasis of gastrointestinal NETs [28], and liver metastasis of illeal carcinoid tumors [26]. Furthermore, there is little data regarding miR-133 in NETs, which warrants further functional evaluation and clinical studies.

### 4.5. miR-223

miR-223 is among the most studied miRNAs in health and disease. Encoded on the X-chromosome, miR-223 has been associated with disease conditions such as sepsis, type 2 diabetes, rheumatoid arthritis, HIV, and inflammatory disorders. Several major transcription factors such as E2F1 and HSP90 are thought to be directly targeted. miR-223 appears to be a myeloid marker, commonly expressed by hematopoietic bone marrow lineage-committed precursor cells that are undergoing neutrophilic differentiation processes [170]. Similarly, cellular differentiation of granulocytes is mediated by miR-223 via retinoic acid and transcription factor C/EB Pα. The importance of miR-223 stemming from its regulation of granulocyte differentiation and proliferation has been known for a while [171]. Additionally, miR-223 regulates multifactorial transcription factor NFI-A, which is a CCAAT-related binding protein (CTF) involved in cellular development and differentiation [172]. Transfection of miR-223 may induce cell cycle arrest in the G2/M phase while also suppressing AKT/ERK signaling, thereby limiting proliferation and invasion, and is thought to decrease the NLR family pyrin domain containing 3 (NLRP3) inflammasome activity, thereby limiting the availability of mature IL-1β/IL-18 and slowing inflammatory processes in various disease conditions, especially those depending on neutrophilic activity [173,174,175]. Signaling through Toll-like receptor (TLR)-9 directly induces miR-223 gene expression in neutrophils through NF-κB, which binds the miR-223 promotor. In turn, data from murine studies have shown that the miR attenuates inflammation by inducing IKK expression, thereby self-limiting the inflammatory function of TLR-9 [176]. In vitro activation of TLR-4 by microbial LPS reduces the expression of miR-223 while promoting pro-inflammatory IL-6 but not TNF through STAT3/NF-κB-dependent signaling [177]. IL-6 additionally negatively affected miR-223 transcription, thereby inducing a positive-feedback loop promoting inflammation. In turn, upregulation of miR-223 limited inflammatory signaling, providing evidence of the direct role in the inflammatory pathway. Similarly, the negative feedback loop between miR-223 and E2F1 in acute myeloid leukemia indicates a regulatory role of miR-223 in cell cycle progression, especially in cells of myeloid origin [178]. In lung cancer as well as cardiac fibrosis models, miR-223 was found to be directly influenced by TGF-β, while TGFβR3 was also found to be a direct target of miR-223 [179,180]. TGF-β signaling promotes downstream PI3K/Akt/mTOR as well as MAPK and ERK signaling, resulting in small mothers against decapentaplegic (SMAD) recruitment and consequent upregulation of differentiation and fibrosis pathways as well as epithelial-to-mesenchymal transition, which could be essential for the development of metastasis [181]. Upregulation of miR-223 promotes apoptosis in lung carcinoma cells dependent on BCL-2/Bid through a direct effect on HSP90B1 [182], while the inhibition of proliferation may be caused by the activity of the tumor suppressor p53, which has also been linked to miR-223 activity [183].

Data regarding miR-223 in NETs remain sparse to date. We were able to show that miR-223 is significantly downregulated in the serum of NET patients, providing possible value as a diagnostic biomarker [30]. Conversely, miRNA levels did not correlate with tumor characteristics, relapse, or overall survival. Lee and colleagues previously evaluated tissue expression of miR-223 in pancreatic NETs, finding no correlation regarding stage or mitotic counts [115].

## 5. Circulating miRNAs for the Diagnosis and Prognosis of NET

The aggressiveness of NETs depends mainly on histological features, such as the extent of proliferation measured by the Ki-67 index [184], neuroendocrine differentiation, and the tumor grading. Subsequent overall survival (OS) may also be affected by additional characteristics, such as the tumor location, hormone production, and metastatic spread [185]. Several studies investigated tissue-specific miRNA expression levels in NETs [185,186], but there are limited data on circulating miRNAs. Due to the simplicity of sampling, low cost, and ability to perform repeated sampling for patient monitoring, blood-based biomarkers have been proposed as an ideal tool for patient stratification, disease surveillance, and treatment evaluation. Such blood-based markers have even been proposed to help define surgical effectiveness or aggressiveness of residual disease [187]. However, identifying robust and effective blood-based biomarkers for NETs has proven challenging. Circulating CgA levels correlate with tumor mass and patient survival [187,188] but have questionable reproducibility, specificity, and sensitivity [187,189]. Recently, there has been considerable interest in the use of miRNAs as a diagnostic tool for NETs as they have proven potential for diagnosis, prediction of therapeutic outcome, and monitoring chemosensitivity of tumors in various cancer types.

## 6. Diagnostic Circulating miRNAs

Comparison of miRNA expression levels in the plasma of patients with small intestinal NETs (siNETs) (*n* = 111) compared to healthy controls (*n* =110) identified a dysregulation of miR-22-3p, miR-21-5p, and miR-150-5p. While miR-22-3p and miR-21-5p, both known to be involved in inflammation and fibrosis, were found to be upregulated in patients with siNETs, miR-150-5p displayed a downregulated pattern [190]. Another study evaluated the circulating expression levels of nine miRNAs, which were previously identified to be dysregulated in tumor tissue of siNET patients [191]. Of these nine miRNA candidates, miR-31, miR-129-5p, miR-133a, and miR-215 were found to be significantly downregulated in the serum of siNET patients as compared to healthy controls, irrespective of the treatment regimen of the patients [192]. miR-7-5p has also been found elevated in the serum of patients with siNET. No correlation with clinical parameters, including tumor stage and UICC stage, was found [27]. Lastly, one other study found miR-125b-5p, miR-362-5p, miR-425-5p, and miR-500a-5p to be upregulated in the serum of siNET patients (*n* = 33) compared to healthy controls (*n* = 14). When these four miRNAs were combined, an AUC of 0.951 was obtained for diagnosis of siNETs [193].

Analysis of the serum of hypergastrinemic patients with autoimmune atrophic gastritis and type 1 gastric NET (gNET) identified significant elevation of miR-222 expression levels. Moreover, treatment of such patients with the CCKR2 agonist netazepide (YF476), which inhibits NET secretion and proliferation, significantly decreased circulating miR-222 compared to healthy controls [194] and it has therefore been proposed as a potential biomarker for gastrin-induced premalignant alterations in the stomach [195].

Several circulating miRNAs have been found to be overexpressed in the serum and tissue of patients with pancreatic NETs (pNETs), including miR-193b as compared to healthy individuals [32] and miR-21 as compared to patients with chronic pancreatitis [186]. Additionally, miR-1290 was found to be overexpressed in patients with pancreatic cancer (*n* = 41) as compared to pancreatic NET (*n* = 18), with an AUC of 0.8. Other miRNAs that could distinguish these two pathologies were miR-628-3p (AUC = 0.68), miR-1825 (AUC = 0.72), miR-550a-5p (AUC = 0.7), miR-1285, and miR-584. Unfortunately, their circulating expression levels were not compared to healthy controls [196]. miRNAs could also be of additional value to already existing blood-based diagnostic markers, as has been shown by the combination of CgA with let-7b-5p, let-7i-5p, miR-143-3p, and miR-30d-5p, which obtained higher diagnostic utility (AUC = 0.752) than application of CgA individually (AUC = 0.672) in patients with pNETs as compared to healthy controls [197].

Limited experimental information is available on the function and target sites of miRNAs in siNET. However, several studies have found that miR-7-5p, miR-182, miR-183, and miR-96-5p are upregulated in sbNETs compared to the normal small intestine, whereas miR-129-5p and miR-133a were found to be downregulated. In addition, miR-182, miR-183, and miR-96 were found to be overexpressed in NET metastases compared to primary tumors [195].

In tumor tissue, blood, and stool samples from patients with colorectal NETs (cNETs), miR-186 was found to be downregulated in comparison to controls. Furthermore, upregulation of PTTG1 was found together with decreased miR-186 expression, suggesting that the upregulation of PTTG1 is induced by loss of miR-186 [198].

Interestingly, significantly lower levels of circulating miR-223 [30] and miR-29 [154] were found in patients with gastroenteropancreatic NET, with primary tumors located in the ileum (*n* = 23), pancreas (*n* = 21), and stomach (*n* = 1), as compared to healthy individuals (*n* = 19), suggesting a circulating mRNA pattern, which overlaps different origins of NET. No correlation of miR-223 levels to clinical or histopathological factors was observed, nor did the miRNA have any prognostic function [30]. However, in the analysis of miR-29 serum levels in patients with NETs, a significant correlation between chromogranin A (CgA) and relative miR-29b levels was found [154].

## 7. Prognostic Circulating miRNAs

In contrast to suggestions that tissue miRNAs can help predict prognosis in NET patients, there are limited data on circulating miRNAs. However, in patients with sbNET, increased circulating levels of miR-21-5p and miR-22-3p and low levels of miR-150-5p were characteristic of metastatic tumors. Consistent with this, low plasma miR-21 levels and high miR-150-5p levels were associated with significantly prolonged overall survival [190].

When comparing SSA-treated siNET patients to healthy controls or SSA-untreated siNET patients, miR-96, miR-182, miR-183, miR-196a, and miR-200a levels were significantly elevated compared to both control groups. SSAs are therefore suggested to significantly affect the expression levels of circulating miRNAs. Interestingly, while these miRNAs showed significantly higher expression after SSA treatment, only miR-200a was elevated in those patients with liver metastases, irrespective of the SSA treatment, thus suggesting that miRNA-200a levels are not dependent on SSAs. As miR-200a has been shown to be involved in the epithelial-to-mesenchymal transition, its importance in tumor progression is suggested [192].

Several studies investigated miRNA expression levels in NETs, but few examined their potential role as prognostic markers. Furthermore, circulating miRNA levels are only weakly associated with tissue miRNA levels, which can themselves also vary widely [185]. However, identification of prognostic markers that predict the outcome of NETs is of utmost importance to ensure the best clinical treatment for these patients. miRNAs as prognostic markers for NETs are now intensively studied, and the most significant results to date have been summarized in detail in the review by Zatelli et al. [185]. In conclusion, studies on the role of circulating miRNAs as prognostic markers in NETs are insufficient. New prospective multicenter studies need to demonstrate the potential of miRNAs in this context before clinical use can be considered.

## 8. Therapeutic Use of miRNAs

As deregulation of miRNA appears to be essential to various diseases’ progression, several studies have been conducted or are currently underway in order to evaluate the efficacy of supplementing anti-miRs or miR-mimics in order to halt disease progression. Commonly used are either viral vectors (adenoviral) or delivery systems such as liposomes (neutral lipid emulsions), dendrimers (conjugated nucleic acids), or PEI (polyethylenimine). In preclinical models, miRNA mimics have been evaluated using miR-34a, targeting K-Ras/p53-induced murine lung adenocarcinoma [199]; miR-143/145, in pancreatic cancer [200]; and anti-miR- 221, successfully improving survival in an HCC model [201]. Several others have evaluated miRNA-directed targeting in preclinical models of atherosclerosis [202], cardiac ischemic injury [203], and diabetic nephropathy [204]. Several clinical studies evaluating the therapeutic potential of targeting miRNA in human disease conditions have been conducted or are currently ongoing. Miravirsen, an anti-miR-122, has shown improvements in patients with ongoing or chronic hepatitis C infection [205]. A phase 1 clinical study evaluating the efficacy of Cobomarsen (MRG-106, anti-miR-155) in various leukemia cases has shown initial improvement of disease condition [206]. On the contrary, a phase 1 clinical trial (NCT01829971) with the miR-34 mimic MRX34 was halted after multiple immune-mediated adverse events, while reductions in advanced tumor stages were shown on the other hand [207]. Several other studies targeting cardiovascular conditions (MGN-1374—miR-15/miR-195), polycystic kidney disease (RGLS4326—miR-17), or fibrotic conditions (Remlarsen—miR-29) are underway and have been recently summarized elsewhere [208], but questions regarding stable compound delivery, possible off-target effects, and reliable target identification have yet to be answered in order to advance miRNA targeting in cancer and specifically in neuroendocrine neoplasia.

## 9. Conclusions and Perspectives

Within this review, we have summarized the available evidence on the role of miRNAs as important drivers of NET development. Using the examples of miR-29, miR-133, and miR-223, each of which regulates important tumor-associated processes such as cell growth and proliferation, we show in great specificity and detail how miRNAs are involved in tumorigenesis. In addition to these miRNAs, we discuss how other miRNAs are also involved in the development of NETs in a variety of ways and control the pathophysiology of NETs in the form of a network. Notably, the deep integration of miRNAs into the pathophysiology of NETs makes them interesting targets for preventing or treating NETs, as recently demonstrated for hepatitis C virus [209,210] therapy and other diseases. Moreover, the favorable chemical properties of miRNAs make them optimal biomarkers to reflect the presence and pathophysiology of tumor diseases such as NETs. The principal suitability of miRNAs as prognostic and/or predictive biomarkers in the context of NETs has been demonstrated for different miRNAs, including miR-21, -29, and -223. Nevertheless, several challenges including sample standardization and normalization of data are yet to be overcome before miRNA-based biomarkers in the setting of NET can be translated into clinical routine.

In conclusion, we have summarized the current knowledge on miRNA use as serum-based biomarkers in patients with NET. We have highlighted opportunities for clinical translation and discussed open issues applicable to future developments.

## Figures and Tables

**Table 1:** Summary of additional potential NET-relevant miRNAs and their possible related functions in apoptosis, proliferation, differentiation, methylation, drug resistance, and immune cell modulation.

miR on Chr.	Potential Functions
**34a/b/c on Chr. 11**	Ubiquitously expressed throughout the organism, with preference for brain, lung, and testes [58] Transcription elements of miR-34 are directly induced through the binding of p53 [58] and ELK-1 [59] as well as FoxO3a [60] and FoxP1, limiting proliferation of regulatory T cells [61] Inverse correlation with anti-apoptotic BCL-2 [62], Survivin, and BRIC5 [63] affects cyclin D1, cyclin-dependent kinase 6 [64], and Cyclin E2 as well as CDK4 [65] Reduces cellular proliferation through MAP3K9 [66] and MAP2K1 [67] Interference with elongation factor E2F3, and possibly a consequential increased activity of cell-death-mediating caspases 3 and 7 [68] Limiting Wnt/ß-catenin through miR-34/p53, reducing invasive capacities in vitro and in vivo [69]
**miR-143/miR-145 on Chr. 5**	Development of smooth muscle tissue, especially in cardiac morphogenesis [70], possibly through TGF-ß signaling Comparative studies have shown downregulation in solid tumors [71] Affects apoptosis through Bcl2/NF-κB and DFF45 [72], proliferation via KRAS/BRAF and YES/STAT1 [73], invasion via MMP13/MMP11 [74], and drug resistance by ABCG2 [75] and DNMT3A-dependent methylation pathways [76,77,78] miR-143 knockdown, while drastically improving the migratory capacity of cancerous cells in a TLR-2-dependent manner [77] Modulation of K-Ras/MEK/ERK signaling through miR-143, as several studies have shown a direct correlation in various models [79,80,81] miR-143 was downregulated in NEN of the small intestine (healthy to control tissue) and was further downregulated in corresponding metastasis [29]; similar results were found for downregulation in insulinoma vs normal pancreatic islet controls [82]
**193a/193b on Chr. 16/17**	Ubiquitously expressed, important for proliferation [83] via cyclin D1, E2F1 [84,85,86], and MAPK/ JNK for G1/S stage transition [87] as well as differentiation and organ development [88] Benefits cell survival, inhibits apoptosis via presenilin 1 and BCL-2 [89,90]; motility, tissue invasion, and angiogenesis via K-Ras/ERBB [91,92,93] LOXL4 benefitting metastasis in breast cancer [94] was also shown to be directly regulated by miR-193 [95] Hypermethylation via DNMT3a is repressed through miR-193 [96] miR-193 corresponding to low levels of TGF-ß dampens the invasive potential of human osteosarcoma cells via Rab27B or SRR [97], similar to SMAD3 in glioma [98]
**375 on Chr. 2**	Impairs beta cell proliferation and fate and regulates insulin secretion [99] and is induced through transcription factors HNF1, INSM1, and Ngn3 [100] Inhibits autophagy through ATG2B [101]/ATG7 [102] and regulates ErbB2, thereby inhibiting mTOR signaling and consequentially promoting apoptosis [103] miR-375 regulates Hippo/YAP signaling, cell proliferation, apoptosis, and contact inhibition [104]; limits in vitro invasiveness [105], and correlates with tumor-promoting YAP1 [106] Wnt5 regulates miR-375 expression, showing direct inverse interaction with miR-375 [107] miR-375 binds TLR-4, limiting inflammagens such as TNF-α, IL-1β, IL-6, and IL-8, possibly through NF-κB and consequential apoptosis [108]

## Data Availability

Not applicable.

## Abbreviations

BCL6	B-cell lymphoma 6
BACH1	BTB and CNC homology 1
CgA	Chromogranin A
FBXO11	F-box only protein 11
FGF2	Fibroblast growth factor
HSP70	Heat shock protein 70
HDL	High-density lipoprotein
HIF-1α	Hypoxia-inducible factor 1α
INoS	Inducible nitric oxide synthases
INF	Interferon
LOXL	Lysyl oxidase-like protein
HMGA2	Mammalian high-mobility-group protein AT-hook 2
MMP	Matrix metalloproteinase
mRNA	Messenger RNA
miRNA	MicroRNA
NET	Neuroendocrine tumor
NF-kb	Nuclear factor kappa-light-chain-enhancer of activated B cells
OS	Overall survival
pNET	Pancreatic NET
PTEN	Phosphatase and tensin homolog
PI3K	Phosphoinositide 3-kinases
AKT	Protein kinase B
RISC	RNA-induced silencing complex
STAT3	Signal transducer and activator of transcription 3
siNET	Small intestinal NET
SSAs	Somatostatin analogues
YAP	Yes-associated protein
